# Supplementary material for: Pharmacological Profile of MP-101, a Novel Non-racemic Mixture of R- and S-dimiracetam with Increased Potency in Rat Models of Cognition, Depression and Neuropathic Pain
Source: Cells. 2022 Dec 13;11(24):4027. doi: 10.3390/cells11244027 (PMC9776800; doi:10.3390/cells11244027)
Supplement: Supplementary file 1 [file cells-11-04027-s001.zip › cells-2056711-SI.pdf]

Pharmacological Profile of MP-101, a Novel Non-racemic Mixture of R- and S-dimiracetam with Increased Potency in Rat Models of Cognition, Depression and Neuropathic Pain

Tiziana Bonifacino [1,2,†], Laura Micheli [3,†], Carola Torazza [1], Carla Ghelardini [3], Carlo Farina [4], Giambattista Bonanno [1,5], Marco Milanese [1,5,*], Lorenzo Di Cesare Mannelli [3,*] and Michael W. Scherz [4]

[1] Department of Pharmacy (DIFAR), Pharmacology and Toxicology Unit, University of Genoa, 16148 Genoa, Italy
[2] Inter-University Center for the Promotion of the 3Rs Principles in Teaching & Research (Centro 3R), 56122 Pisa, Italy
[3] Department of Neuroscience, Psychology, Drug Research and Child Health, NEUROFARBA-Pharmacology and Toxicology Section, University of Florence, 50139 Florence, Italy
[4] Metys Pharmaceuticals c/o Novaremed AG, 4051 Basel, Switzerland
[5] IRCCS, Ospedale Policlinico San Martino, 16132 Genoa, Italy
* Correspondence: marco.milanese@unige.it (M.M.); lorenzo.mannelli@unifi.it (L.D.C.M.)
† These authors contributed equally to this work.

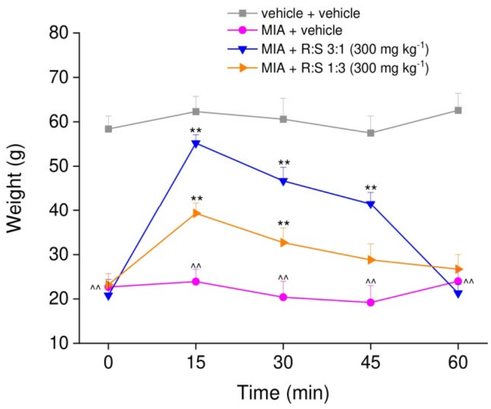

Supplementary Figure S1. Evaluation of the effects of R:S 3:1 and R:S 1:3 against MIA-induced mechanical hyperalgesia. Sodium monoiodoacetate (MIA) 2 mg/25 μL was injected into the tibio-tarsal joint of rats on day 1. Tested mixtures were administered acutely 14 days after MIA administration at the dose of 300 mg kg^{-1} *per os*. Control animals were treated with vehicles. Mechanical hyperalgesia was measured on the ipsilateral paw by the Paw pressure test. Results are expressed as mean ± S.E.M. of 8 rats analysed in 4 different experimental sets. Statistical analysis was one-way ANOVA followed by Bonferroni's post hoc comparison. ^^P<0.0 vs vehicle + vehicle treated animals; **p<0.01 vs MIA + vehicle treated animals.

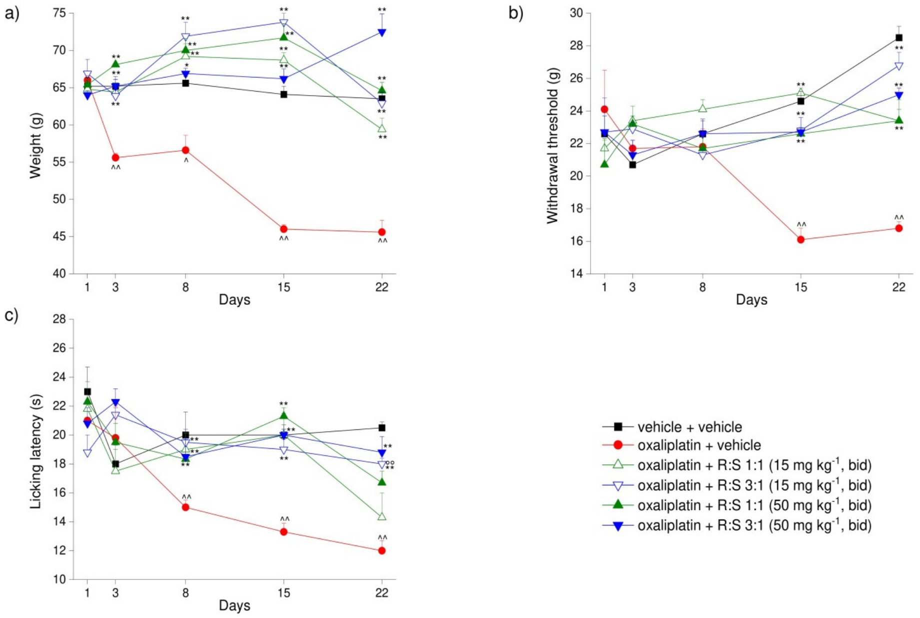

Supplementary Figure S2. Evaluation of the acute effects of R:S 1:1 and R:S 3:1 against oxaliplatin-induced neuropathic pain. Oxaliplatin (2.4 mg kg^{-1} i.p.) was administered on days 1-15, for a total of 11 injections. R:S 1:1 and R:S 3:1 were dissolved in 1% CMC and were daily *per os* administered from day 1 until day 22 (15 and 50 mg kg^{-1} bid, in the morning and in the evening). Control animals were treated with vehicles. In the graphs are represented the behavioral measurements performed 1 h after treatments. The pain relieving effects of R:S 1:1 and R:S 3:1 was assessed by a) Paw pressure test, b) von Frey test and c) Cold plate at the indicated time points. Each value represents the mean ± S.E.M. of 6 rats. Statistical analysis was one-way ANOVA followed by Bonferroni's post hoc comparison. ^ $p < 0.05$; ^^ $p < 0.01$ vs vehicle + vehicle; *p<0.05 and **p<0.01 vs oxaliplatin + vehicle; °°p<0.01 vs oxaliplatin + R:S 1:1 15 mg kg^{-1}.
